# A Mechanically Resilient and Tissue‐Conformable Hydrogel with Hemostatic and Antibacterial Capabilities for Wound Care

**DOI:** 10.1002/advs.202303651

**Published:** 2023-09-13

**Authors:** Jae Park, Tae Young Kim, Yeonju Kim, Soohwan An, Kyeong Seok Kim, Minkyong Kang, Soo A Kim, Jayoung Kim, Joonseok Lee, Seung‐Woo Cho, Jungmok Seo

**Affiliations:** ^1^ School of Electrical and Electronic Engineering Yonsei University Seoul 03722 Republic of Korea; ^2^ LYNK Solutec Inc. Seoul 03722 Republic of Korea; ^3^ Department of Biotechnology Yonsei University 50–1 Yonsei‐ro, Seodaemun‐gu Seoul 03722 Republic of Korea; ^4^ Department of Chemistry Hanyang University Seoul 04763 Republic of Korea; ^5^ Department of Medical Engineering College of Medicine Yonsei University Seoul 03722 Republic of Korea

**Keywords:** antibacterial, hemostasis, hydrogels, tissue adhesives, wound dressing

## Abstract

Hydrogels are used in wound dressings because of their tissue‐like softness and biocompatibility. However, the clinical translation of hydrogels remains challenging because of their long‐term stability, water swellability, and poor tissue adhesiveness. Here, tannic acid (TA) is introduced into a double network (DN) hydrogel consisting of poly(vinyl alcohol) (PVA) and poly(acrylic acid) (PAA) to realize a tough, self‐healable, nonswellable, conformally tissue‐adhesive, hemostatic, and antibacterial hydrogel. The TA within the DN hydrogel forms a dynamic network, enabling rapid self‐healing (within 5 min) and offering effective energy dissipation for toughness and viscoelasticity. Furthermore, the hydrophobic moieties of TA provide a water‐shielding effect, rendering the hydrogel nonswellable. A simple chemical modification to the hydrogel further strengthens its interfacial adhesion with tissues (shear strength of ≈31 kPa). Interestingly, the TA also can serve as an effective hemostatic (blood‐clotting index of 58.40 ± 1.5) and antibacterial component, which are required for a successful wound dressing. The antibacterial effects of the hydrogel are tested against *Escherichia coli* and *Staphylococcus aureus*. Finally, the hydrogel is prepared in patch form and applied to a mouse model to test in vivo biocompatibility and hemostatic performances.

## Graphical abstract

A tannic acid‐incorporated double network hydrogel offers toughness, self‐healing capability, conformal tissue interfacing ability, hemostatic effects, and antibacterial properties. The hydrogel can be utilized as a mechanically resilient and tissue‐conformable wound dressing patch owing to its multifunctionality. The hydrogel wound dressing patch effectively seals the wound site and rapidly induces hemostasis.

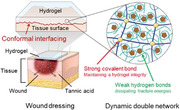

## 1 Introduction

Numerous surgical procedures involve wound dressings to stop bleeding and leakage, provide an antibacterial barrier, and ensure tissue healing. Clips, staples, and sutures are considered the gold standard for wound closure methods currently used in the clinical field. However, there are unmet challenges, such as insufficient leakage prevention, uncontrolled hemostasis, and bacterial infection during suturing.^[^ ^1^ ^]^ Moreover, these methods are time‐consuming and require highly trained personnel, which is undesirable in emergency cases. These methods can also damage soft tissues, thereby worsening treatment and delaying tissue repair. Easy placement and removal of wound dressings without causing tissue damage are required in emergency medical situations. Accordingly, bioadhesive materials have been developed as alternatives to staples and sutures, as they have the potential to reduce surgical time and minimize additional trauma.^[^ ^2^ ^]^ They are easy to use, biocompatible, and exhibit good tissue adhesion properties. There are two primary types of bioadhesive: injectable glues and adhesive patches. Currently, bioadhesive glues, including fibrin glue and cyanoacrylate, are primarily used clinically.^[^ ^3^ ^]^ Their liquid‐like properties allow them to seamlessly penetrate the irregular rough geometries of tissue surfaces. Then, the penetrated glues are cured to ensure cohesion. However, they often fail to provide reliably strong adhesion strengths against tissues, and cyclic deformation of the tissue results in their fracture because the cured glues are brittle.^[^ ^4^ ^]^ Synthetic polymer‐based glues may involve the leakage of chemicals into the surrounding tissues before curing, potentially causing toxicity. Furthermore, covering large wound areas with bioadhesive glues is arduous. Bioadhesive patches, such as polyurethane films, offer reliable tissue adhesion and flexibility to adapt to the movements of tissues and cover large wound areas. However, they cannot precisely comply with irregularly shaped tissue surfaces, as their Young's moduli exceed that of soft tissues.^[^ ^5^ ^]^ Thus, they can be easily detached from the body during dynamic movements. Critically, commercial bioadhesives are designed to be monofunctional and offer only mechanical adhesion.^[^ ^2b^ ^]^ The clinical application of bioadhesives faces numerous challenges, such as bacterial infection and uncontrolled hemostasis. Efforts are needed to design a multifunctional bioadhesive that can effectively address these problems and thereby completely replace sutures and staples in the medical field.

Recently, advances in multifunctional hydrogels have been achieved in various biomedical fields.^[^ ^6^ ^]^ The main advantage of hydrogels is their tissue‐like softness (moduli of ≈100 kPa), which allows for mechanical matching with irregularly shaped soft tissues. Hydrogels have been extensively studied as bioadhesives owing to their soft interfacing abilities.^[^ ^7^ ^]^ Moreover, the incorporation of antibacterial or hemostatic agents into hydrogels provides functionalities for effectively addressing medical issues.^[^ ^8^ ^]^ However, hydrogels are still far from clinical translation. Soft hydrogels are typically brittle and mechanically weak; therefore, they can easily fracture in dynamic and harsh tissue environments.^[^ ^9^ ^]^ Critically, the tissue adhesion strength of hydrogels is low because they generally utilize intermolecular forces, such as hydrogen bonds, electrostatic interactions, and van der Waals forces.^[^ ^10^ ^]^ Although covalent bonds have also been adopted for hydrogel–tissue adhesion, the brittle hydrogels cannot endure the high covalent bonding strength; therefore, a cohesive failure easily occurs under small stresses.^[^ ^11^ ^]^ To improve the mechanical properties of hydrogels, double network (DN)‐based tough hydrogels have recently been investigated.^[^ ^12^ ^]^ DN hydrogels consist of long polymer chains and sacrificial chains. When strain is applied to the hydrogel, the sacrificial chains dissipate the fracture energies, whereas the long chains maintain the integrity of the hydrogel. The DN strategy also allows hydrogels to covalently bond to tissues and endure large strains, preventing cohesive failures.^[^ ^13^ ^]^ Although DN hydrogels are durable, mechanical damage may yet occur over the long term. Self‐healing hydrogels utilizing reversible cross‐linking networks have emerged as long‐lasting bioadhesives that can reconstruct damaged structures.^[^ ^14^ ^]^ However, achieving hydrogels that are simultaneously self‐healing and tough is challenging because the high polymeric chain density of DN hydrogels restricts the dynamic reconfiguration for self‐healing.^[^ ^15^ ^]^ Furthermore, introducing additional polymer chains increases Young's modulus and viscous properties of hydrogels, hindering their seamless soft interfacing with tissues. Viscoelastic properties enabling hydrogels to adapt to rough tissue surfaces are required to implement conformal interfacing with tissues.^[^ ^16^ ^]^ Moreover, the inherent water‐swellability of hydrogels is a critical issue in their medical applications, and tough hydrogels are especially susceptible to water swelling because of the increased number of additional polymer chains with hydrophilic functional groups.^[^ ^17^ ^]^ Additionally, the increased polymer chain density of DN hydrogels elevates the osmotic pressure, further promoting water uptake.^[^ ^18^ ^]^ A new design strategy for hydrogels is required to realize rational multifunctionality for clinical applications.

In this study, we have developed a nonswellable hydrogel bioadhesive that is mechanically durable and capable of conformal interfacing with tissues. We introduced tannic acid (TA), a natural polyphenol compound containing abundant catechol and pyrogallol groups, to a DN hydrogel. Several other polyphenol compounds such as dopamine and tyramine have been explored for bioadhesives; among them, TA possessing 25 hydroxyl groups in its single molecule, actively interacts within hydrogel network through hydrogen bonds. The hydroxyl‐rich nature of TA enables to realize a dynamic network within the hydrogel, which can effectively dissipate energy. The effective energy dissipation imparts viscoelasticity and toughness to the hydrogel. The viscoelasticity allows precise fitting of the hydrogel to irregular tissue surface geometries. When the hydrogel adheres to the tissue surfaces, stress relaxation occurs and gradually adapts to the surface by minimizing the mechanical mismatch. Furthermore, simple chemical modification of the hydrogel further offers strong covalent adhesion to tissue surfaces. As the hydrogel also exhibits high toughness, it can endure high covalent adhesion strength without the cohesive failure. The dynamically TA‐crosslinked hydrogel can also rapidly self‐heal after mechanical damage due to its reversible crosslinking, enabling the hydrogel to deal effectively with mechanical damage and fractures. Additionally, the hydrophobic moieties of TA render the hydrogel resistant to water swelling so that it can retain its mechanical properties even under highly hydrated conditions. Combining these features ensures the mechanical durability of the hydrogel adhesive, allowing it to endure harsh and dynamic tissue environments. Notably, TA also serves as a hemostatic and antibacterial component; both properties are crucial for wound dressing applications. The hydroxyl‐rich TA is known to actively interact with blood proteins such as globulin, albumin, and other coagulation factors.^[^ ^19^ ^]^ This promotes blood coagulation near the hydrogel, indicating that the hydrogel has a potent hemostatic ability and is suitable for wound‐dressing applications. A few hemostatic hydrogels have been reported; however, they require a long time (>90 s) with steady pressure for hemostasis, which is undesirable in emergency cases; the hydrogel developed in this study enables rapid blood coagulation (within 10 s) upon application to the wound site. In addition, TA interrupts the biological activity of bacteria so that the hydrogel exerts antibacterial effects.^[^ ^20^ ^]^ Considering that bacterial infections are a common threat in clinical applications, the antibacterial property is advantageous for the clinical translation of the hydrogel. To demonstrate the in vivo efficiency of the hydrogel, it was fabricated in a patch form, and its in vivo biocompatibility and hemostatic ability were verified. Introducing TA into DN hydrogels offers multiple functionalities required for rational wound‐dressing bioadhesives.

## 2 Results and Discussion

### 2.1 Design and Fabrication of the Multifunctional Hydrogel

**Figure 1:**
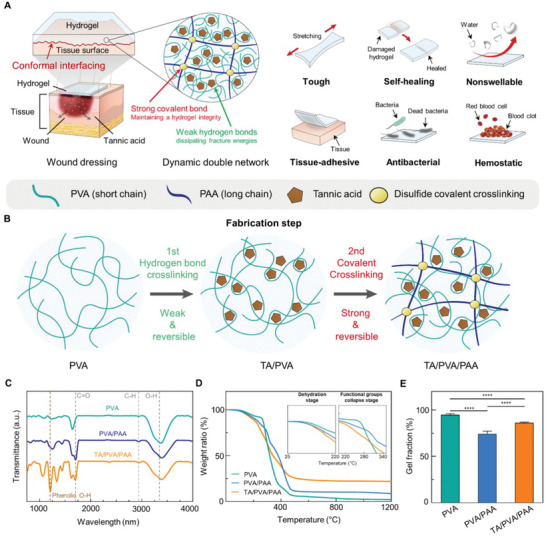
Multifunctional hydrogel for wound dressing patch. A) Schematic illustrations of a DN hydrogel wound dressing patch and its multifunctionality. B) Fabrication of the developed DN hydrogel. C) FT‐IR spectra of hydrogels. D) Thermogravimetric analyses for hydrogels. E) Gel fraction measurements for each hydrogel sample (*n* = 3: *n* is the sample size for each group).

**Figure** **1**A shows a schematic of the TA‐incorporated DN hydrogel used for wound dressing, along with depicting the hydrogel's multifunctionality. The DN consists of poly(vinyl alcohol) (PVA) and poly(acrylic acid) (PAA), which dissipate energy and maintain hydrogel integrity, respectively. In particular, the short PVA chains are dynamically crosslinked via hydrogen bonds with TA, and long PAA chains are covalently crosslinked through N,N′‐bis(acryloyl) cystamine‐containing disulfide bond. The DN structure ensures the toughness of the hydrogel, thereby enabling it to endure external stresses occurring in the tissue environment. Interestingly, the hydrogel can also be conformally attached to tissue surfaces, as the dynamic interactions that occur between the DN network and TA result in the hydrogel's viscoelasticity. Functionalization of PAA with 1‐Ethyl‐3‐(3‐dimethylaminopropyl) carbodiimide (EDC) and N‐hydroxysuccinimide (NHS) activation further enable strong covalent bonding with tissue surfaces.^[^ ^21^ ^]^ In addition, TA offers multiple additional functionalities, including self‐healing, nonswellability, and antibacterial and hemostatic effects. The reversible nature of hydrogen and disulfide bonds allows the autonomous, fast self‐healing of the hydrogel.^[^ ^22^ ^]^ Thus, the hydrogel can restore its original structure and intended functionalities even after mechanical damage that may occur upon application to tissues. Because the hydrogel is nonswellable, it can retain its volume and mechanical properties even in humid environments. These properties contribute to ensuring the mechanical stability of the hydrogel so that it can robustly adhere to the tissue and maintain its functionalities. Moreover, the multiple catechol and pyrogallol phenolic groups of TA exhibit hemostatic and antibacterial effects.^[^ ^19^ ^]^ Therefore, the hydrogel can effectively induce blood coagulation and simultaneously prevent severe bacterial infections. The multifunctionality of the hydrogel can address the unmet issues associated with medical wound dressings.

The hydrogel is prepared using a two‐step fabrication process (Figure 1B). First, we introduce the TA as a reversible crosslinker to form a dynamic network. TA, which has 25 hydroxyl groups in a single molecule, provides abundant hydrogen bonds to the hydrogel network and realizes a dynamic network. Acrylic acids are then interpenetrated into the TA/PVA network, followed by their polymerization and disulfide crosslinking. Each fabrication step is chemically demonstrated by using X‐ray photoelectron spectroscopy (XPS) (Figure S1, Supporting Information). After introducing the TA into the PVA, a C═O peak (≈288 eV) appears, indicating the presence of TA molecules. The interpenetration of PAA is confirmed by the increased intensity of the C─C peak (≈284 eV). The hydrogels are further analyzed using Fourier transform infrared (FT‐IR) spectroscopy, thermogravimetric analyzer (TGA), and measuring gel fractions. We compare the TA/PVA/PAA hydrogel with a single‐network hydrogel, PVA, and a conventional DN hydrogel, PVA/PAA, to verify the effects of TA on the DN hydrogel. Figure 1C shows the FT‐IR spectra for each hydrogel sample. All samples exhibit broad C─H and O─H peaks at 2938 and 3364 cm^−1^, respectively. The C═O peak at 1700 cm^1^ appears for the PVA/PAA and TA/PVA/PAA because of the presence of carboxyl and carbonyl groups in each sample. The phenolic O─H peak at 1320 cm^−1^ is observed for TA/PVA/PAA, implying that the TA is well incorporated into the sample. TGA is subsequently performed on dried hydrogel samples to determine their compositions and thermal stabilities (Figure 1D). First, no obvious decrease in the weight ratio of hydrogels is observed at 37 °C, indicating the thermal stability of the hydrogels under human body conditions. The TGA of polymer samples typically shows a triphasic curve characterized by the sequential occurrence of three distinct events: the removal of adsorbed water, the collapse of functional groups, and the subsequent decomposition of the main chain.^[^ ^23^ ^]^ All samples demonstrate slight decreases in their weight ratio until 220 °C, which corresponds to the dehydration of adsorbed water molecules on the hydroxyls. A steeper decrease in the weight ratio of the TA/PVA/PAA sample before 220 °C indicates the high water‐capturing ability of the abundant hydroxyl groups in TA. The PVA/PAA and TA/PVA/PAA samples exhibit lower onset temperatures for the secondary decreasing step than the PVA sample. This is because PAA initiates its decarboxylation at 220 °C,^[^ ^24^ ^]^ while PVA begins a collapse of a hydroxyl group and partial decomposition at 280 °C. The TA/PVA/PAA sample begins its sharpest decrease at the 220 °C phase as the decomposition of oxygen‐containing groups in the TA overlaps with PAA decomposition;^[^ ^25^ ^]^ these curves suggest that each constituent is successfully introduced into the hydrogel. Next, we examine the crosslinking efficiency of the hydrogels by measuring their gel fractions (Figure 1E). PVA, crosslinked by semi‐crystalline regions, shows the highest gel fraction of ≈94.85. The conventional double network PVA/PAA exhibits a far lower gel fraction (≈74.39%). This is because PAA interpenetration reduces the crystallinity density of PVA.^[^ ^26^ ^]^ The TA/PVA/PAA hydrogel shows a higher gel fraction of ≈86.05%, surpassing that of PVA/PAA. TA also reduces the crystallinity of PVA, but it serves as a stable crosslinker to ensure the gel network.^[^ ^27^ ^]^ Although the gel fraction of the TA/PVA/PAA was lower than that of the PVA, a gel fraction greater than 85% is considered a stable gel network.^[^ ^28^ ^]^ Compared to PVA/PAA DN hydrogel, the TA/PVA/PAA with the higher crosslinking can exert more efficient energy dissipations for toughening. Lastly, the water retention of the TA/PVA/PAA hydrogel was characterized, because maintaining moisture conditions on the wound sites is vital for wound dressing (Figure S2, Supporting Information). The water content of the hydrogel at equilibrium was ≈45.29%, and the water was retained over 24 h, suggesting that the hydrogel is well designed for wound dressing. The overall results confirm the successful introduction of TA into the DN hydrogel and the stability of the resultant hydrogel.

### 2.2 Mechanical Durability of the Hydrogel

**Figure 2:**
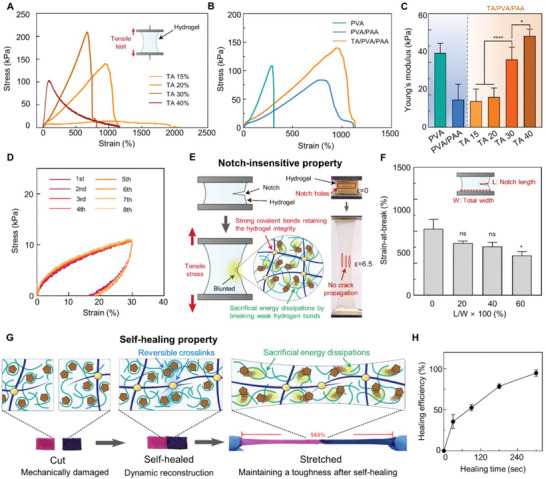
Mechanical durability of the hydrogel. A) Tensile test for the TA/PVA/PAA hydrogels with varying concentrations of TA. B) Tensile test comparing PVA, PVA/PAA, and TA/PVA/PAA. C) Young's moduli of hydrogel samples (*n* = 3: *n* is the sample size for each group). D) Cyclic loading‐unloading tests for the TA/PVA/PAA hydrogel (*n* = 3: *n* is the sample size for each group). E) Schematic illustration describing the notch‐insensitive property of the hydrogel. F) Strain at break values of the hydrogels with varying notch lengths. G) Schematic illustration and optical photographs showing the self‐healing behavior of the hydrogel. H) Self‐healing efficiencies of the hydrogel as a function of time (*n* = 3: *n* is the sample size for each group). Statistical analysis was carried out with an unpaired *t*‐test (^*^ *p* < 0.05, ^**^ *p* < 0.01, ^***^ *p* < 0.001, and ^****^ *p* < 0.0001; ns: no significant difference).

Hydrogels consisting of water are as soft as tissues but are simultaneously brittle and weak. The mechanical weakness of hydrogels is a critical issue that must be addressed to realize practical hydrogel bioadhesives. As tissues undergo frequent movements and deformations, hydrogels cannot endure the external stresses applied by the tissue environment. In particular, slight mechanical damage to the hydrogels easily results in crack propagation upon the occurrence of small strains in the hydrogels.^[^ ^29^ ^]^ The designed DN hydrogel exhibits toughness based on the fracture energy dissipation mechanism. The toughening property also renders the hydrogel notch‐insensitive; therefore, it does not fracture even in the presence of mechanical damages or defects. Notably, the hydrogel can restore its initial mechanical structure and properties based on the reversible crosslinking within its network. This self‐healing property further ensures the long‐term stability of the hydrogel. TA plays a critical role as it provides effective energy dissipation for toughness, and reversible crosslinking for rapid self‐healing.^[^ ^29^, ^30^ ^]^ Therefore, we first optimized the mechanical properties of the TA/PVA/PAA hydrogel by varying the TA concentration (**Figure** **2**A; Figure S3, Supporting Information). Increasing the TA concentration up to 30% w/v elevated the tensile strength and reduced the strain‐at‐break. This suggests that a higher concentration of the TA forms a densely crosslinked and entangled network with higher mechanical strength. However, excessive amounts of TA result in aggregation, leading to network heterogeneity and reduced stretchability.^[^ ^31^ ^]^ These aggregations hinder efficient sacrificial energy dissipation, which allows for a larger strain. Increasing the concentration of the TA over 30% w/v leads to a reduction in the tensile strength of the hydrogel because of the heightened presence of TA coagulants. We selected a TA concentration of 20% w/v as it exhibits high stretchability up to ≈1109% strain and a sufficient tensile strength of ≈119 kPa, which can endure loads occurring in tissues. We then compared the mechanical properties of the TA‐incorporated DN hydrogel with those of a single‐network hydrogel, PVA, and a conventional DN hydrogel, PVA/PAA (Figure 2B). The PVA showed the highest tensile strength of ≈108 kPa; however, its strain was limited to less than ≈310%. The crosslinking through crystalline regions within PVA offers high tensile strength, but PVA fails to allow large strain as it is a single network that does not have dissipative components. Both PVA/PAA and TA/PVA/PAA, which are DN hydrogels, allowed larger strains than PVA. In particular, TA/PVA/PAA exhibited higher strain and strength, far exceeding those of PVA/PAA. These results imply that TA effectively serves as a dissipative component within the DN hydrogel and reinforces its toughness. Figure 2C shows Young's moduli of PVA, PVA/PAA, and TA/PVA/PAA with varying concentrations of TA. For the TA/PVA/PAA sample, the modulus increases with increasing TA concentration. This corresponds with rubber elasticity theory, which states that denser crosslinking results in a higher modulus.^[^ ^32^ ^]^ Specifically, the modulus is significantly elevated at TA concentrations of 30%. All samples possess moduli similar to that of soft tissues (e.g., liver, 4.0–6.5 kPa; cardiac muscle, 8 kPa; skeletal muscle, 5–170 kPa; skin, ≈100 kPa).^[^ ^33^ ^]^ The optimized TA/PVA/PAA hydrogel with 20% w/v TA exhibits a modulus of 18.65 ± 3.78 kPa, suggesting its potential to seamlessly match with tissue surfaces. We further examined the dissipative properties of the hydrogel by carrying out a cyclic tensile‐compressive test (Figure 2D). The inner area of the curve represents the amount of dissipated energy. The cyclic tensile‐compressive experiment for the TA/PVA/PAA hydrogel reveals a large number of repeatable energy dissipations. In contrast, the conventional double network hydrogel PVA/PAA shows a far smaller area under the curve, and it substantially decreases after the first cycle (Figure S4, Supporting Information). In particular, the hydrogel can repeatedly dissipate energy because of its dynamic network reconstructing broken TA cross‐linkages. The curve shows similar stress‐dissipation behaviors over five cycles. These results indicate that the hydrogel can repeatedly exert its toughness in tissue environments where external stresses frequently occur.

Next, we demonstrated the notch‐insensitive property of the optimized hydrogel. Figure 2E shows a schematic illustration of the hydrogel's notch‐insensitive property and photographs showing a stretched TA/PVA/PAA hydrogel with notch holes. Generally, hydrogels are vulnerable to notches, in that applying a slight strain to a notched hydrogel immediately causes crack propagation. However, based on the energy dissipation mechanism, our hydrogel endures large strains even in the presence of a notch. As the notched hydrogel is elongated, the TA and PVA sacrificially dissipate stress, while the long PAA chain maintains the integrity of the hydrogel, thereby preventing crack propagation. Tensile tests were conducted on the notched hydrogel samples with varying notch lengths (Figure 2F; Figure S5, Supporting Information). The notched samples were elongated over 750% strain, indicating that no crack propagation occurred during the stretching. The notch of the sample was blunted instead of propagating crack upon stretching the hydrogels (Figure S6, Supporting Information). The reversibly crosslinked dynamic network also endows the hydrogel with a self‐healing ability. Specifically, the damaged networks can be rapidly reconstructed via abundant hydrogen bonds owing to the hydroxyl‐rich nature of TA. Figure 2G shows a schematic illustration and a photograph of the self‐healing process of the hydrogel. Two pieces of cut hydrogel rapidly self‐heal based on the TA‐mediated reversible crosslinking. Notably, the main chain of PAA can also be re‐crosslinked because it is covalently bonded through a reversible disulfide crosslink. Both the main and dissipative chains of the DN hydrogel can restore their initial structure. The self‐healing of TA/PVA/PAA restores not only mechanical connections but also restores the initial mechanical properties of the hydrogel. Thus, the self‐healing hydrogel can be highly stretched. The self‐healing efficiency of the hydrogels was examined as a function of time (Figure 2H; Figure S7, Supporting Information). This efficiency was calculated by comparing the tensile strengths of the initial and self‐healed samples. The hydrogel reconstructed its state by up to ≈40% after a healing time of 30 s and completely healed by up to 100% after 300 s. Also, the TA/PVA/PAA hydrogel revealed no significant differences in Young's modulus before and after the self‐healing, indicating that the hydrogel completely restore the initial mechanical properties (Figure S8, Supporting Information). Conventional autonomous self‐healing hydrogels require a long time (over a dozen hours) for complete self‐healing.^[^ ^34^ ^]^ Although several rapidly self‐healing materials exist, they are usually in a liquid state with low viscosities.^[^ ^35^ ^]^ A liquid‐like behavior ensures high polymer chain mobility, enabling quick network reconstruction.^[^ ^29^ ^]^ However, their high flowability and low viscosity hamper the practical applications of hydrogels. TA renders the DN hydrogel mechanically stable and capable of rapid self‐healing. To confirm the influence of TA on the double network hydrogel, additional investigations were performed on PVA, PVA/PAA, and alginate/poly(acrylamide) (PAAm) hydrogels without TA. For the conventional double network tough hydrogels, PVA/PAA and alginate/PAAm, they can be highly elongated (Figure S9, Supporting Information); however, they are not able to self‐heal. The damaged pieces of PVA, PVA/PAA, and alginate/PAAm samples revealed a lack of self‐healing capability, even after prolonged periods of contact (Figure S10, Supporting Information). This implies that TA with high‐density hydroxyls plays a substantial role in dynamic network reconstruction and self‐healing function. Investigations of the mechanical properties and self‐healing ability of the hydrogel suggest that it possesses robust durability to bear external loads and can withstand mechanical damage.

### 2.3 Interfacial Stability of the Hydrogel Against Tissues

**Figure 3:**
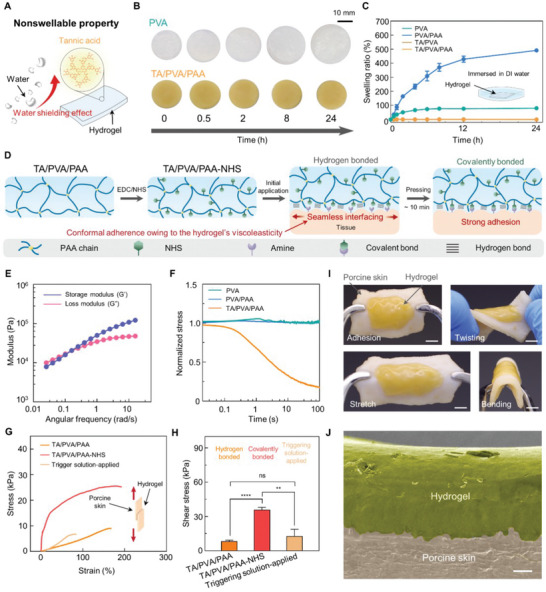
Interfacial stability of the hydrogel. A) Schematic illustration depicting nonswellable property of the hydrogel. B) Sequential photographs of the PVA and TA/PVA/PAA hydrogels immersed in water. C) Swelling ratio measurement of each hydrogel sample as a function of time (*n* = 3: *n* is the sample size for each group). D) Schematic illustration of the adhesion process of the hydrogel. E) Rheological analysis of the TA/PVA/PAA hydrogel. F) Stress‐relaxation test for each hydrogel sample. G) Lap shear test characterizing the adhesion strength of the hydrogel against porcine skins. H) Shear strength of the hydrogel without‐ and with NHS ester (*n* = 3: *n* is the sample size for each group). I) Photographs of the hydrogel conformally attached to a porcine skin (scale bars, 5 mm). J) SEM image of the hydrogel adhered to a porcine skin surface (scale bar, 100 µm).

The robust and conformal interfacing ability of bioadhesives must be achieved to tightly seal wounds and prevent leakages. For example, they should have strong adhesion strength to tissue surfaces and mechanically comply with dynamically deforming tissues, as humans frequently move. In other words, bioadhesives must form conformal interfaces with tissues. The soft nature of hydrogels is beneficial for conformal interfacing; however, their brittleness and poor adhesion strength pose a challenge to achieving their long‐term stability in dynamic tissue environments. In addition, swelling is a critical issue when hydrogels interface with tissues. Hydrated tissue surfaces lead to water penetration into the hydrophilic hydrogel networks, thereby degrading the mechanical properties of the interfaced hydrogel. The developed hydrogel exhibits nonswellability because of the hydrophobic regions in TA. Moreover, the chemically modified hydrogel can robustly adhere to tissue surfaces via covalent bonds and accommodate various deformations of tissues without being detached or fractured. The high toughness of the hydrogel allows covalent bonding with tissues without undergoing a cohesive failure. As the hydrogel is also viscoelastic, it can gradually minimize mechanical mismatch with tissues and comply with the dynamic movements of tissues. First, we verified the nonswellability of the hydrogel. **Figure** **3**A shows a schematic of the nonswellability of the hydrogel. The TA within the hydrogel consists of hydrophobic moieties; thus, it exerts a water‐shielding effect. Figure 3B shows optical photographs of volume changes in the PVA and TA/PVA/PAA hydrogels upon water immersion. The volume of the PVA hydrogel gradually expanded with increasing immersion time. In contrast, the TA/PVA/PAA hydrogel retained its volume even after 24 h of immersion. The swelling ratios of hydrogels with varying components were also measured as a function of immersion time (Figure 3C). No increase in mass change was observed in the TA/PVA and TA/PVA/PAA groups, whereas PVA and PVA/PAA showed elevation in their mass. The swelling ratio of PVA/PAA (≈500%) was far higher than that of PVA (≈177%) owing to increased hydrophilic polymer chains in the PVA/PAA. The swelling experiment suggests that TA plays a crucial role in nonswellable properties. The developed hydrogel will effectively prevent the degradation of its mechanical properties by blocking water penetration upon attachment to tissues. We then investigated the mechanical compliance of the hydrogel to tissues. The viscoelasticity enables the hydrogel to penetrate the rough and irregular geometries of tissue surfaces precisely. Further chemical modification of the PAA in the hydrogel with EDC/NHS endows it with the capacity to covalently adhere to interfaced tissues. Figure 3D shows a schematic illustration of the process of hydrogel–tissue interfacing. Before adhering the hydrogel to tissues, the carboxyl groups of the PAA within the hydrogel are chemically modified with EDC/NHS activation. The resulting TA/PVA/PAA‐NHS can form covalent bonds with primary amines on tissue surfaces. Upon application of the hydrogel to a tissue surface, its viscoelasticity enables it to seamlessly interface with the rough tissue surfaces, thereby strengthening their mechanical interlocking. In addition, the conformal adhesion of the hydrogel increases the contact area between the hydrogel and tissue, potentially allowing efficient covalent bond formation between the NHS and primary amines on the tissue surfaces. After pressing the hydrogel for 10 min, strong covalent bonding occurs, resulting in a robust tissue–hydrogel interface. We characterized the viscoelastic properties of the hydrogel by performing a rheological analysis and examining its stress‐relaxation behavior. Figure 3E shows the storage (*G′*) and loss moduli (*G′′*) of the hydrogel as a function of angular frequency. The *G′* was higher than the *G′′* above the frequency of 0.1 rad s^−1^; however, both values were within similar ranges, indicating that the hydrogel is a viscoelastic solid. Notably, *G′* was gradually declined as the angular frequency decreases. This is attributed to the stress relaxation behavior of the hydrogel, which is the decrease in stress in response to strain in the hydrogel.^[^ ^36^ ^]^ A lower angular frequency provides more time to decrease the energy stored by stress relaxation. The stress‐relaxation test was performed to characterize the stress‐relaxation behavior of the hydrogel (Figure 3F). The stresses of each sample were measured as a function of time while applying a constant strain of 15%. The PVA and PVA/PAA hydrogels exhibited dominant elastic behavior with no decrease in their initial stresses. In contrast, the TA/PVA/PAA hydrogel exhibited rapid stress relaxation, with its stress decreasing to half of its initial stress in only a few seconds. Stress‐relaxing behavior is beneficial for conformal interfacing, as it enables the reduction of mechanical mismatch at the hydrogel–tissue interface and renders the hydrogel precisely fit to irregular surfaces. These viscoelastic properties of the hydrogel ensure its seamless attachment and mechanical compliance to rough tissues. NHS ester modification to the hydrogel enables it to form covalent bonding with tissue surfaces containing primary amine groups.^[^ ^3^ ^]^ Critically, the toughness of the hydrogel allows it to endure strong covalent adhesion, thereby preventing cohesive failure. Figure S11 (Supporting Information) shows a photograph of the NHS‐functionalized hydrogel adhered to porcine skin. No cohesive failure occurred even after stretching the adhered hydrogel. The strong adhesion of wound dressings often results in tissue damage and patient discomfort during the process of wound dressing removal.^[^ ^2b^ ^]^ Applying the triggering solution containing sodium bicarbonate and L‐glutathione provides painless removal of the covalently adhered TA/PVA/PAA‐NHS (Figure S12, Supporting Information).^[^ ^37^ ^]^ The basic pH of the solution neutralizes the carboxyl groups of the PAA chains, and sodium ions ionically couple with the carboxyl groups, thereby depriving the hydrogen bonding ability. The L‐glutathione reduced disulfide crosslinks of the PAA network, leading to cleavage of the hydrogel network from the tissue surface. We characterized the adhesion strength of the TA/PVA/PAA, NHS‐functionalized TA/PVA/PAA, and trigger solution‐applied TA/PVA/PAA‐NHS to porcine skin (Figure 3G,H). The lap shear test revealed that covalently bonded TA/PVA/PAA‐NHS hydrogel exhibited the highest shear strength of ≈35.98 kPa. In contrast, the TA/PVA/PAA mainly relied on hydrogen bonds, and the trigger solution‐applied TA/PVA/PAA‐NHS exerted ≈8.28 and ≈12.72 kPa, respectively. The result indicates that the hydrogel can be robustly adhered to tissue surfaces and also can be easily removed without tissue damage. The stress‐displacement curve for the lap shear test for TA/PVA/PAA‐NHS shows significant displacements of 30 mm, which corresponds to a ≈200% elongation of the adhered hydrogel. This implies that the hydrogel effectively dissipates energies while pulling the covalently bonded porcine skin substrates; thus, a large strain on the hydrogel occurs without its fracture. In contrast, the application of trigger solution to TA/PVA/PAA‐NHS resulted in a lower strain value of ≈61%, which is attributed to the cleavage of disulfide crosslinks within PAA. Figure 3I shows photographs of hydrogel‐interfaced porcine skin with various deformations. The hydrogel is stably adhered to the porcine skin upon stretching, twisting, and bending, suggesting it can accommodate diverse deformations of tissues. Finally, we visualized the hydrogel‐porcine skin interface via scanning electron microscopy (SEM) to observe the conformal interfacing (Figure 3J). The image shows the seamless attachment of the hydrogel to the rough geometry of the skin tissue surface. Overall, the hydrogel conformally and covalently adheres to tissue surfaces without mechanical mismatches. Conformal attachment ensures mechanical compliance of the hydrogel with deforming tissues. Thus, the hydrogel can tightly seal the wound site even under frequent patient movement. The demonstrated robust interfacing ability of the hydrogel with tissues enables it to serve as an effective wound dressing.

### 2.4 Biocompatibility and Hemostatic Property of the Hydrogel

**Figure 4:**
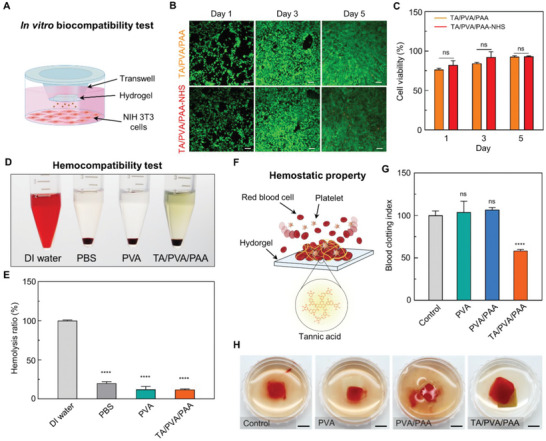
Biocompatibility and hemostatic effects of the hydrogel. A) Schematic illustration describing the in vitro biocompatibility test. B) Fluorescence microscopy images of live/dead stainings (green: live, red: dead) on NIH 3T3 cells cultured with hydrogels (scale bars, 200 µm). C) Cell viability calculated from the live/dead assay (*n* = 3: *n* is the sample size for each group). D) Optical image of hemolysis test on hydrogels (*n* = 4: *n* is the sample size for each group). E) Hemolysis ratio of each group. F) Schematic illustration describing the hemostatic effects of the hydrogel. G) Blood clotting index value for each group (*n* = 4: *n* is the sample size for each group). H) Photographs of blood clotting on each hydrogel (scale bars, 5 mm). Statistical analyses were conducted with an unpaired *t*‐test (^*^ *p* < 0.05, ^**^ *p* < 0.01, ^***^ *p* < 0.001, and ^****^ *p* < 0.0001; ns: no significant difference).

TA has been shown to offer unique mechanical properties to DN hydrogels. Interestingly, TA is also beneficial for hemostasis, which is essential for wound dressing.^[^ ^38^ ^]^ A primary step in the wound healing process is hemostasis; therefore, uncontrolled blood coagulation is often the cause of wound dressing failure. Although a few hemostatic hydrogels have been reported, they require a prolonged application time (at least over 1.5 min) and pressure to achieve hemostasis.^[^ ^39^ ^]^ The hydroxyl‐rich TA actively reacts with blood proteins such as globulin, albumin, and other coagulation factors, thereby promoting effective hemostasis.^[^ ^38^ ^]^ The developed hydrogel containing abundant TA induces rapid hemostasis on adjacent wounds; this effectively prevents blood loss and ensures unhindered tissue repair. Combining the hemostatic function of the hydrogel with its conformal interfacing ability, the hydrogel is highly advantageous for application in wound dressings. The seamless attachment maximizes the contact area between the hydrogel and the wound site, thereby further enabling effective hemostasis. Before exploring the hemostatic function of the hydrogel, we examine its biocompatibility with fibroblasts to confirm its safety for skin tissues. We also evaluated the cytotoxicity of TA/PVA/PAA‐NHS to demonstrate the biocompatibility of the chemical modification with NHS. Biocompatibility tests were performed by incubating NIH 3T3 fibroblasts with the hydrogels, followed by a live/dead assay using a transwell system (**Figure** **4**A). A live/dead assay for PVA and PVA/PAA revealed no cytotoxicity issues (Figure S13A, Supporting Information). Figure 4B,C shows the fluorescence images and quantified cell viabilities for the live/dead assay for TA/PVA/PAA, respectively. After 1, 3, and 5 days of exposure, neither the hydrogels without NHS nor the hydrogel with NHS exhibited any significant toxicity toward fibroblasts. A direct submerge method was also employed to examine the biocompatibility of the TA/PVA/PAA hydrogel (Figure S13B, Supporting Information). Therefore, the chemically modified hydrogels can be readily applied to skin tissues. Next, we investigate the hemostatic properties of the hydrogels. First, the blood compatibility of the hydrogel was evaluated by performing a hemolysis assay.^[^ ^40^ ^]^ The hydrogel‐conditioned erythrocyte solutions were examined to verify whether the destruction of red blood cells and the release of hemoglobin occur. The liquid was centrifuged to separate hemoglobin from the supernatant. The supernatant was subsequently spectrophotometrically analyzed. We also tested deionized (DI) water and (Phosphate‐buffered saline) PBS as positive (obvious hemolysis) and negative control (no hemolysis), respectively. Figure 4D shows a photograph of the centrifuged samples. Except for the positive control of the DI water, the supernatants were clear, indicating the absence of hemoglobin. Figure 4E shows the spectrophotometric analysis of the supernatants from each group. The PVA and TA/PVA/PAA hydrogels exhibit a hemolysis ratio of ≈11%, which is not significantly different from that of the negative control, i.e., the PBS group with no hemolysis. These results suggest that the hydrogel is hemocompatible and can be used as a blood‐contacting material. We then explored the hemostatic effects of hydrogels. Figure 4F shows a schematic illustration describing hemostasis on the TA/PVA/PAA hydrogel. The TA within the hydrogel actively interacts with red blood cells, platelets, coagulation factors, and blood proteins. Specifically, the phenolic hydroxyl groups of TA activate the clotting factor XII, thereby promoting rapid blood clotting.^[^ ^41^ ^]^ The blood clotting index (BCI) was calculated to evaluate the hemostatic properties of the hydrogel (Figure 4G). A lower BCI indicates a high hemostatic performance, and the TA/PVA/PAA hydrogel showed a BCI of 58.40 ± 1.5 which is approximately two times lower than those of the control, PVA, and PVA/PAA groups. A clearly coagulated blood clot was observed adjacent to the TA/PVA/PAA hydrogel surface, whereas the other groups showed poor hemostasis with the spreading of the blood components within the DI water (Figure 4H). These results suggest that the developed hydrogel is biocompatible and offers effective hemostatic ability due to the presence of TA. Thus, the hydrogel can serve as a rational wound‐dressing patch that meets the urgent need for wound treatment.

### 2.5 Antibacterial Performances of the Hydrogel

**Figure 5:**
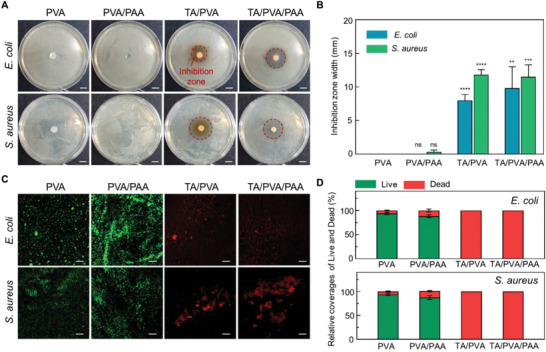
Antibacterial performances of the hydrogel. A) Representative photographs of the hydrogels and bacteria colonies of *E. coli* and *S. Aureus* (scale bars, 1 cm). Red dashed lines indicate inhibition zones. B) Diameter of inhibition zones formed near the hydrogels after 12 h of incubation (*n* = 3: *n* is the sample size for each group). C) Fluorescent images of live/dead bacteria (green: live, red: dead) of *E. coli* and *S. aureus* incubated with the hydrogels (scale bars, 200 µm). D) Quantitative analysis of adherent live and dead bacteria on the samples after 12 h of incubation in a growth medium (10^5^ bacterial mL^−1^) (*n* = 3: *n* is the sample size for each group).

Bacterial infection is another major concern in wound treatment as it leads to severe inflammation and hinders tissue healing. Furthermore, bacterial growth and colonization on bioadhesive materials can alter their intended properties. Although sterilization methods and strict hygiene protocols for preventing infections have been established in clinics, the risk of infection remains. The current method of reducing infections in the clinical field is antibiotic administration; however, prolonged dosage of antibiotics results in antibiotic resistance in bacteria. Strategies to develop antibacterial bioadhesive materials are desirable to ensure the inhibition of infection. TA is known to exhibit antibacterial effects; therefore, the hydrogel itself effectively prevents bacterial infections. The antibacterial properties of TA are attributed to its astringency and antioxidative properties.^[^ ^42^ ^]^ While TA actively binds with various biomolecules, it exerts astringency, which induces complexation with bacterial enzymes. Furthermore, TA affects bacterial membranes by inhibiting oxidative phosphorylation within them. This oxidative phosphorylation inhibition notably reduces bacterial activity; therefore, the TA‐incorporated DN hydrogel can be applied to wound dressings without infectious side effects. We examined the antibacterial effects of the hydrogel against representative gram‐negative and gram‐positive infectious pathogenic bacteria, *Escherichia coli* (*E. coli*) and *Staphylococcus aureus* (*S. aureus*) respectively. These bacteria are selected as they are common hospital bacteria that present a threat in surgeries. An inhibition zone test was conducted to evaluate the antibacterial performance of the hydrogels. **Figure** **5**A shows representative optical microscopy images of each hydrogel sample after 24 h of cultivation with *E. coli* and *S. aureus*. TA/PVA and TA/PVA/PAA showed obvious inhibition zones, whereas no inhibition zones were observed near PVA and PVA/PAA. A significant increase in the inhibition zones of the TA‐containing samples was statistically demonstrated by measuring the diameters of the inhibition zones (Figure 5B). The average diameters of the inhibition zones for TA/PVA and TA/PVA/PAA were ≈8.03 ± 0.7 and 9.83 ± 2.59 mm, respectively, in *E. coli*‐cultured agar plates. Similarly, the mean diameters of the inhibition zones for TA/PVA and TA/PVA/PAA were 11.8 ± 0.62 and 11.5 ± 1.47 mm, respectively, in *S. aureus*‐cultured agar plates. We further performed a bacterial viability assay using fluorescent probes, SYTO 9, and propidium iodide. Figure 5C shows the fluorescent images for each hydrogel group cultured with *E. coli* and *S. aureus*; green signifies live bacteria and red indicates bacteria that have lost their viability. The PVA and PVA/PAA hydrogel groups exhibited predominantly green fluorescence, whereas the TA/PVA and TA/PVA/PAA hydrogels revealed predominantly red fluorescence. This confirms that the presence of TA molecules plays a critical role in the antibacterial effects of the hydrogel. The coverage of the fluorescence for live and dead staining was quantified, and the TA‐containing samples revealed significantly decreased bacterial viability (Figure 5D). These results imply that the developed hydrogel can inhibit bacterial growth, thereby substantially reducing the risk of infection.

### 2.6 In Vivo Biocompatibility and Hemostatic Ability of the Hydrogel Patch

**Figure 6:**
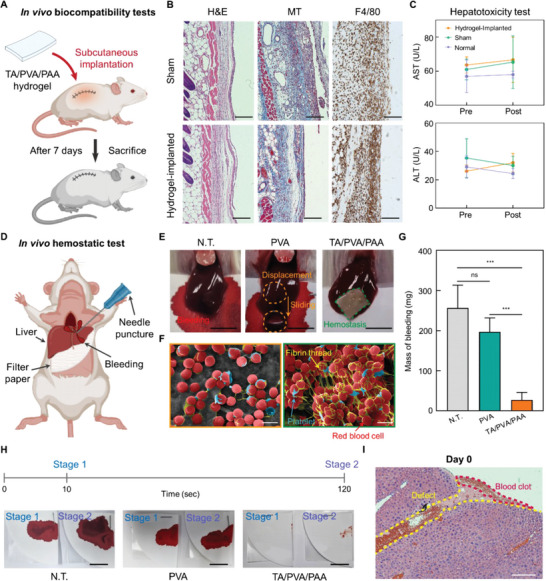
In vivo biocompatibility and hemostatic performances of the TA/PVA/PAA patch. A) Schematics of subcutaneous implantation of TA/PVA/PAA hydrogel into the backs of ICR mice. B) Histological analyses after 7 days of implantation: H&E, nuclei were stained blue, and the cell cytoplasm were stained pink; MT, collagen was stained in blue, nuclei were stained in black, and the cytoplasm was stained in red; F4/80, macrophage marker stained in brown (scale bars, 100 µm). C) Hepatotoxicity evaluation with measurement of serum level of ALT and AST (*n* = 3: *n* is the sample size for each group). D) Schematic describing a hemostatic experiment in a mouse model. E) Optical photographs of the N.T., PVA, and TA/PVA/PAA groups in a liver hemorrhage model (scale bars, 5 mm). F) SEM images of PVA and TA/PVA/PAA hydrogel surface after hemostasis experiment (scale bars, 5 µm). G) The accumulated amount of bleeding mass in the filter papers for N.T., PVA, and TA/PVA/PAA groups (*n* = 4: *n* is the sample size for each group). H) Optical photographs of the filter papers that absorbed bleeding blood from the damaged liver at the time points of 10 and 120 s (scale bar, 10 mm). I) H&E staining of the liver tissue where hemostasis was induced by the TA/PVA/PAA patch (scale bars, 200 µm). Statistical analysis was performed with an unpaired *t*‐test (^*^ *p* < 0.05, ^**^ *p* < 0.01, ^***^ *p* < 0.001, and ^****^ *p* < 0.0001; ns: no significant difference).

The TA/PVA/PAA hydrogel was demonstrated to possess desirable multifunctionality for wound dressings. To further evaluate in vivo efficacy of the hydrogel, we fabricated the hydrogel in a wound‐dressing patch form and tested it using a mouse model. We first examined the in vivo biocompatibility of the patch by performing histological analyses and hepatotoxicity assays. The TA/PVA/PAA patches were subcutaneously implanted into mice, which were sacrificed 7 days after the surgery (**Figure** **6**A). A sham group of incised mice without patch implantation was also established. Figure S14 (Supporting Information) shows photographs of the cutaneous skin of the patch‐implanted mice right after implantation and after 7 days. No abnormal cutaneous skin reactions related to local acute inflammation, such as swelling, blistering, infiltration, erythema, or ulceration, were observed at the implantation sites. To further examine the biocompatibility of the patch through histological analysis, implanted patch‐adjacent tissues were stained with hematoxylin and eosin (H&E), Masson's trichrome (MT), and immunohistochemical staining using anti‐F4/80 antibodies (Figure 6B). The H&E and MT stainings revealed no signs of toxicity in the tissues surrounding the patch.^[^ ^43^ ^]^ The F4/80 staining detects macrophages (brown), thereby indicating the level of inflammatory response. There were no significant differences observed in the macrophage density between the sham and patch groups. A hepatotoxicity assay was performed to confirm biocompatibility (Figure 6C). In addition to the sham and patch groups, a normal group with no incision is included in this test. The levels of serum alanine aminotransferase (ALT) and aspartate aminotransferase (AST) were compared before implantation and 7 days after implantation. No significant differences were observed in the levels of ALT and AST across the normal, sham, and patch groups. The in vivo biocompatibility tests indicate that the TA/PVA/PAA patch is biocompatible; thus, it can be readily applied as a wound‐dressing patch.

The hemostatic capability of the TA/PVA/PAA patch was evaluated by comparison with that of a PVA hydrogel patch as a negative control. The PVA and TA/PVA/PAA patches were placed on damaged bleeding mouse livers; the damage was induced by an 18‐gauge needle puncture (Figure 6D,E) shows optical photographs of the non‐treated (N.T.) and patch‐applied livers. The amount of bleeding observed in the PVA group was similar to that of the N.T. group; by contrast, the TA/PVA/PAA patch exhibited substantially reduced bleeding owing to its hemostatic properties. In addition, the TA/PVA/PAA patch stably adhered to the damaged site even upon blood burst pressure, whereas the PVA patch was easily displaced from the damaged site by hepatic hemorrhage pressures. Figure S15 (Supporting Information) shows photographs of the hydrogel‐attached liver right after the adhesion of the hydrogel and after 7 days of the application. The TA/PVA/PAA stably adhered to the liver surface. Notably, the hydrogel was seamlessly integrated with the liver tissue after 7 days, owing to the biocompatibility and conformal interfacing ability of the hydrogel. The robust tissue‐interfacing ability of the TA/PVA/PAA patch with its hemostatic effects allows for a practical wound‐dressing patch. The blood clotting induced by the TA/PVA/PAA patch was further analyzed using SEM (Figure 6F). Relatively few red blood cells (RBC), platelets, and fibrin meshes were observed on the PVA patch surface. No fibrin thread formations were observed on the PVA, suggesting no hemostasis occurred. By contrast, dense RBCs, platelets, and fibrin threads, indicating obvious blood coagulants, were observed on the TA/PVA/PAA patch surfaces. Bleeding was also quantified by measuring the amount of blood on the filter paper at the damaged sites (Figure 6G). The TA/PVA/PAA group showed a significantly lower blood mass than the N.T. and PVA groups. Importantly, the TA/PVA/PAA patch induces hemostasis within 10 s of application. Figure 6H shows photographs of the filter papers applied at 10 and 120 s after applications of PVA and TA/PVA/PAA. The N.T. and PVA groups revealed a high amount of blood on the filter paper at 10 and 120 s, implying no hemostasis occurred. On the other hand, almost no blood was observed on the filter paper of the TA/PVA/PAA group, even after only 10 s. Given that existing hemostatic bioadhesives require more than 90 s with steady pressure, the hydrogel possesses the potential to be reliably applied to clinics including emergency cases. Histological analyses were additionally performed to confirm blood clotting (Figure 6H; Figure S16, Supporting Information). The TA/PVA/PAA‐applied livers were retrieved right after and after 7 days of the applications and stained with H&E and toluidine blue (TB). The histological images showed a clear blood clot structure in the coagulated region. Furthermore, no noticeable physiological abnormalities were observed in the TA/PVA/PAA patch‐treated liver tissues. After 7 days, the physically injured defect region formed by the needle puncture was shortened and filled by cells, suggesting that the tissue repair was not hindered owing to the effective hemostasis. These in vivo results suggest that the developed patch can rapidly and effectively induce hemostasis in vivo. Hence, the hydrogel patch holds great potential to fulfill the unmet needs of wound‐dressing hydrogels in the clinic.

## 3 Conclusion

We have developed a multifunctional hydrogel for a wound‐dressing patch by incorporating TA into a PVA/PAA‐based DN hydrogel. Although conventional DN hydrogels possess high toughness, their high polymer chain density limits their other functionalities, including self‐healing, nonswellability, and tissue‐like softness. TA enables the DN hydrogel to achieve these functionalities as the abundant hydroxyl groups of TA dynamically interact with the hydrogel network. TA‐mediated reversible crosslinking endows the hydrogel with softness, viscoelasticity, and rapid self‐healing properties. The reversible crosslinking of the developed hydrogel offers an immediate reconstruction of the hydrogel network after mechanical damage while also providing sacrificial energy dissipation for enhanced toughness. Hence, toughness, softness, and self‐healing can be achieved simultaneously. In addition, the hydrophobic moieties of TA render the hydrogel non‐swellable; thus, it can retain its mechanical properties even in highly hydrated tissue environments. Interestingly, the hydrogel, possessing a dynamic network, exhibits viscoelasticity, which enables conformal interfacing with the irregular geometries of soft‐tissue surfaces. The stress relaxation behavior of the hydrogel ensures its adaptability to tissue surfaces by gradually reducing the mechanical mismatch with the tissues. EDC/NHS activation of the hydrogel further strengthens tissue adhesion. By combining the aforementioned mechanical properties, the hydrogel can be robustly and conformally attached to wound sites. Furthermore, TA serves to impart hemostatic and anti‐bacterial properties. We demonstrate the rapid hemostasis‐inducing ability of the hydrogel patch in vivo. Given that delayed and uncontrolled blood clotting and bacterial infections are major concerns in wound dressing applications, the developed hydrogel has great potential as a wound dressing patch. We envision that the TA‐incorporated DN hydrogel can address various issues in wound treatments.

## 4 Experimental Section

### Fabrication of TA/PVA/PAA Hydrogel

The 20% w/v TA (Sigma–Aldrich) was added into a PVA solution (20% w/v in DI water, *M*_w_ 89 000–98 000) and stirred for 2 h at 90 °C. The obtained TA/PVA hydrogel was frozen at −20 °C for 8 h, followed by thawing at room temperature for 3 h. The TA/PVA was then sequentially dried at 37 and 100 °C for 1 h each. To interpenetrate a PAA network into the TA/PVA, the dried TA/PVA film was immersed in an acrylic acid solution (30% w/w acrylic acid, 0.03% w/w N,N′‐bis(acryloyl) cystamine, and 0.15% w/w 2,2′‐azobis(2‐methylpropionamidine) dihydrochloride in DI water) for 2 h. The soaked film was then heated at 70 °C for 30 min to obtain the TA/PVA/PAA hydrogel. To endow the adhesive property to the hydrogel, an EDC/NHS activation was performed on the PAA chains. The TA/PVA/PAA hydrogel was immersed in a 2‐(N‐morpholino) ethanesulfonic acid (MES) buffer containing EDC (0.5% w/w) and NHS sodium salt (0.25% w/w) for 5 min at room temperature.

### Characterizations

Chemical analyses of the hydrogel were conducted using XPS (K‐alpha, Thermo, UK) and FT‐IR (Vertex 70, Bruker, USA). Freeze‐dried hydrogel samples were prepared for XPS analysis. A circular sampling area of the freeze‐dried samples with a diameter of 400 µm was analyzed using an XPS spectrometer equipped with an X‐ray source of the Al K‐α line. FT‐IR spectra were obtained in the spectral range of 4000–650 cm^−1^ The thermal stability and compositions of the freeze‐dried hydrogel samples were confirmed using a TGA (SDT Q600, TA instruments, USA). The test was conducted under continuous nitrogen flow with a heating rate of 10 ˚C min^−1^, with a thermal range of 30−1400 ˚C. A gel fraction test was performed to characterize the hydrogel crosslinking efficiency. The hydrogels were prepared with a sample size of 10 × 10 mm^2^, followed by drying them at 37 ˚C for 24 h. After the hydrogel reached a constant minimum weight, the dried samples were weighed (*W*_o_) and immersed in DI water for 24 h. The swollen samples were dried at 37 ˚C for 24 h and their masses were reweighed (*W*_e_). Finally, the gel fraction was calculated using the following equation: gel fraction (%) = *W*_e_/*W*_o_ × 100%. The water retention test was performed at room temperature and a humidity level of 55–60%. The weight of the hydrogel samples was measured at each time point, and the water retention was calculated using the equation: water retention (%) = (*W*_s_–*W*_d_)/*W*_e_–*W*_d_. *W*_e_ and *W*_s_, are the weights of the hydrogel at equilibrium and at the time points, respectively. *W*_d_ represents the weights of the dried hydrogel.

### Mechanical Characterizations

Tensile tests of the hydrogels were performed using a mechanical testing machine (MultiTest 2.5‐DV, Mecmesin, UK) equipped with a 50 N load cell at a tensile rate of 50 mm min^−1^. The sample size for the tests was ≈10 × 10 × 1 mm^3^. The tensile‐compressive test was performed at a speed of 10 mm min^−1^. Self‐healing efficiency was calculated by comparing the ultimate tensile strengths of the samples before and after self‐healing. The rheological analyses were conducted using a rheometer (MCR102, Anton Paar, Austria). The hydrogel samples were sandwiched between 25 mm‐diameter‐parallel plates with a gap of ≈1 mm. The storage and loss moduli were measured in a frequency range of 0.1–100 rad s^−1^ at a constant strain of 1%. To characterize the stress relaxation behavior of the hydrogels, their stress was measured as a function of time upon the application of a constant strain of 15%.

### Adhesion Force Tests

The adhesion strength of TA/PVA/PAA‐NHS hydrogel to porcine skin was tested by performing a lap shear test. The test was conducted using a mechanical test machine (MultiTest 2.5‐DV, Mecmesin, UK) equipped with a 50 N load cell. The hydrogel was sandwiched between two skin substrates with dimensions of 76 × 26 × 1 mm^3^ (adhesion area of 15 × 20 mm^2^). The tensile speed of 10 mm min^−1^ was applied. The measured maximum force was divided by the adhesion area to calculate the shear strength.

### In Vitro Biocompatibility

NIH 3T3 fibroblast cells (2.25 × 10^5^ cells mL^−1^) were seeded in a transwell plate with DMEM (Dulbecco's modified Eagle Medium) supplemented with 10% bovine calf serum and 1% penicillin‐streptomycin. The hydrogels were placed in the transwells and incubated with the NIH 3T3 cells. Cell viability assay was performed every 1, 3, and 5 days using a Live/Dead kit (L3224, Invitrogen, USA) following the kit instruction. A laser scanning confocal microscope (LSM 700, Carl Zeiss, Germany) was used to visualize the viability of the cells. The viability was calculated from the stained images using ImageJ software.

### In Vitro Hemostatic Assays

To test the hemostatic ability of the hydrogel, female, Institute of Cancer Research (ICR) mice were anesthetized, and the whole blood was collected. The collected blood was decalcified using sodium citrate. Before the experiment, the decalcified blood was treated with calcium chloride. The volume of 1 mL of the blood was loaded on the hydrogel placed in the plate well. The volume of 5 mL of DI water was added to the well to collect uncoagulated blood. Then, the absorbance of the DI water was measured at 540 nm using a spectrophotometer.

### Hemolysis Assay

Fresh horse blood (MB‐H1880, Kisan Bio Inc., Korea) was used for the hemolysis test. The horse blood was centrifuged using a cryogenic centrifuge at 1500 rpm for 10 min. Then, the supernatant was aspirated and washed with PBS three times. The hydrogel samples with 10 × 10 mm^2^ size were submerged in PBS for 72 h at 37 °C. The volume (0.1 mL) of centrifuged erythrocytes was added to 0.9 mL of the hydrogels‐conditioned PBS. The erythrocyte solutions were centrifuged at 12 000 rpm for 15 min to examine the hemolysis. The absorbances of the supernatants were measured at 540 nm through a spectrophotometer.

### In Vitro Antibacterial Assay

All materials were sterilized in an autoclave at 121 °C for 15 min before use. Gram‐negative *E. coli* and Gram‐positive *S. aureus* were precultured in Luria Bertani broth (LB,10 mL) in an incubator at 37 °C, for 24 h. To determine the concentrations of the stock cultures, the plate counting technique was employed using a 3 m Petri film count plate. The inhibition zone test was performed using bacteria suspension diluted to a concentration of 10^5^ CFU mL^−1^. A volume of 100 µL of a diluted bacterial suspension was spread onto the surface of an LB Agar High Salt Plate, and a hydrogel sample was placed on top of the plate. The test groups were divided as follows: 1) negative control, 2) PVA, 3) PVA/PAA, 4) PVA/TA, and 5) PVA/PAA/TA. Each group of plates was incubated in a chamber at 37 °C for 12 h. After incubation, a ruler was used to determine the diameter of the inhibition zone. The inhibition zone was calculated as follows:

(1) $$\mathrm{Inhibition}\;\mathrm{zone}\;\mathrm{width}\;\;\left(\mathrm{mm}\right)=\;\left(d_{2}-d_{1}\right)/2$$

where *d*_1_ is the diameter of the hydrogel sample and *d*_2_ is the diameter of the antibacterial zone. All experiments were conducted four times. After the inhibition zone test, the bacteria that grew on the surface of each group of hydrogel samples were stained using the Live & Dead staining kit for 15 min in dark conditions (LIVE/DEAD BacLight Bacterial Viability Kit, ThermoFisher, China). Following the instructions provided, SYTO 9 and propidium iodide (PI) were used to stain the live and dead bacteria, respectively. The stained biofilm was then visualized using laser scanning confocal microscopy.

### Hydrogel Implantation into Mouse Subcutaneous Skin

Animal experiments followed the guidelines for the care and use of laboratory animals and were approved by the Institutional Animal Care and Use Committee (IACUC) at Yonsei University (authorization number; IACUC‐A‐202305‐1675‐01). Seven weeks old female ICR mice were obtained from Orient Bio Inc. (Gyeonggi‐do, South Korea) and maintained under semi‐specific pathogen‐free conditions with a 12‐h light/dark cycle after a quarantine period. The mice were anesthetized with ketamine and xylazine. Then, the mice's left back was shaved, and a 10 mm incision was made on the skin to create a subcutaneous pocket. The TA/PVA/PAA hydrogels were implanted into the subcutaneous pockets, and the incisions were closed with a surgical suture thread. After implantations, the mice were housed for 7 days and monitored to check if discomfort or body weight loss existed.

### Histological Analyses

The mice were euthanized after 7 days of implantations, and the hydrogel samples along with the surrounding tissue were collected. The samples were then fixed in 4% paraformaldehyde overnight and embedded in paraffin wax. Thin sections (3–5 µm thickness) were prepared from each sample and mounted onto slides for histological staining. H&E staining, which stains the nuclei blue and cytoplasm pink, was used to examine the inflammatory response. MT staining, which stains collagen blue, cytoplasm red, nuclei dark red/purple, and cytoplasm red/pink, was used to assess collagen formation and organization. Images were obtained using a bright field inverted fluorescence microscope (IX81, Olympus, Japan). The collagen density was determined by measuring the coverage of blue‐pixel in the images of MT staining. Immunostaining with an F4/80 antibody (#70 076, Cell Signaling Technology, USA) was conducted to identify macrophages in the tissues after 7 days of implantation. Antigen retrieval, endogenous peroxidase blocking, and serum blocking were carried out on the collected sections before immunostainings. The sections were incubated with F4/80 antibody overnight at 4 °C. The incubated sections were washed with PBS and incubated with horse radish peroxidase‐labeled goat antirabbit antibodies (GB23303, Servicebio, China) at room temperature for 60 min in a dark room. The sections were then washed three times and incubated with a freshly prepared diaminobenzidine (DAB) chromogenic reagent kit (G1211, Servicebio, China). The nuclei of the sections were counterstained with hematoxylin staining solution (G1004, Servicebio, China) for 3 min and then washed with water. Macrophages stained by the DAB reagent appear brown, while other nuclei stained with hematoxylin appear blue.

### In Vivo Hemostatic Tests in Mouse Model of Liver Incision

The hemostatic effects of the hydrogel were examined in a mouse model with liver hemorrhage using a previously reported method.^[^ ^42^ ^]^ Seven weeks old female ICR mice were obtained from Orient Bio Inc. and were anesthetized. An incision was made in the abdominal area, and sterilized filter papers were placed beneath the liver. Bleeding was induced using an 18 G needle, and the damaged area was immediately covered with either PVA or TA/PVA/PAA hydrogel. The filter papers were replaced after 10 s because of an initial burst release of bleeding caused by the needle. The blood‐absorbed filter papers were collected at time points of 10 and 120 s. The collected filter papers were then weighed to measure the amount of blood. A control group of untreated mice was also used in this study.

### Statistical Analysis

Statistical analyses were conducted with GraphPad Prism software (GraphPad Software Inc., USA) and Origin Pro (Origin Lab Corporation, USA). The differences between experimental groups were analyzed using an unpaired *t*‐test. The levels of significance are as follows: ^*^ *p* < 0.05; ^**^ *p* < 0.01; ^***^ *p* < 0.001; ^****^ *p* < 0.0001; ns: not significant.

## Conflict of Interest

The authors declare no conflict of interest.

## Supporting information

Supporting Information

## Data Availability

The data that support the findings of this study are available from the corresponding author upon reasonable request.
