# Supplementary material for: A Mechanically Resilient and Tissue‐Conformable Hydrogel with Hemostatic and Antibacterial Capabilities for Wound Care
Source: Adv Sci (Weinh). 2023 Sep 13;10(30):2303651. doi: 10.1002/advs.202303651 (PMC10602564; doi:10.1002/advs.202303651)
Supplement: Supplementary file 1 — Supporting Information [file ADVS-10-2303651-s001.pdf]

ADVANCED SCIENCE
Open Access

Supporting Information

for *Adv. Sci.*, DOI 10.1002/advs.202303651

A Mechanically Resilient and Tissue-Conformable Hydrogel with Hemostatic and Antibacterial Capabilities for Wound Care

*Jae Park, Tae Young Kim, Yeonju Kim, Soohwan An, Kyeong Seok Kim, Minkyong Kang, Soo A Kim, Jayoung Kim, Joonseok Lee, Seung-Woo Cho and Jungmok Seo**

Supporting Information

A Mechanically Resilient and Tissue-conformable hydrogel with Hemostatic and Antibacterial Capabilities for Wound Care

Jae Park[†], Tae Young Kim[†], Yeonju Kim, Soohwan An, Kyeong Seok Kim, Minkyong Kang, Soo A Kim, Jayoung Kim, Joonseok Lee, Seung-Woo Cho, Jungmok Seo[*]

[†]These authors contributed equally to this work.

J. Park, T. Y. Kim, Y. Kim, M. Kang, S. A. Kim, J. Seo
School of Electrical and Electronic Engineering, Yonsei University, Seoul 03722, Republic of Korea.

J. Park, J. Seo
LYNK Solutec inc., Seoul, 03722 Republic of Korea.

S. An, S. W. Cho
Department of Biotechnology, Yonsei University, 50-1 Yonsei-ro, Seodaemun-gu, Seoul 03722, Republic of Korea.

K. S. Kim, J. Lee
Department of Chemistry, Hanyang University, Seoul 04763, Republic of Korea.

J. Kim
Department of Medical Engineering, College of Medicine, Yonsei University, Seoul 03722, Republic of Korea

E-mail: jungmok.seo@yonsei.ac.kr

Keywords: hydrogels, hemostasis, wound dressings, antibacterial, tissue adhesives

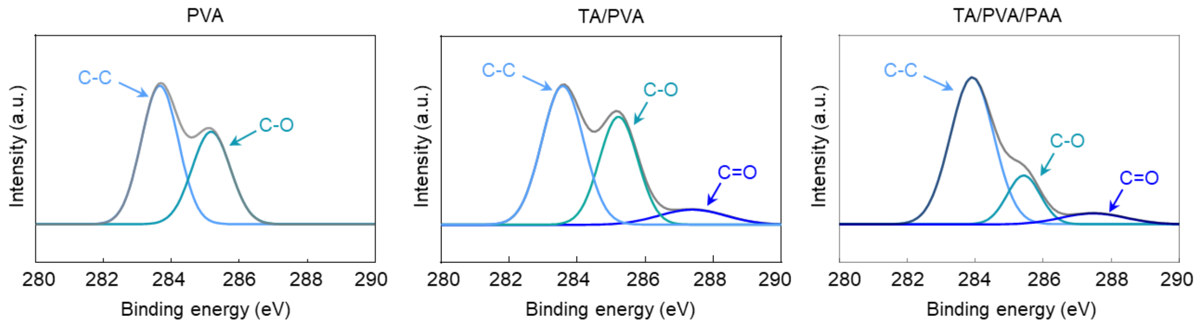

Figure S1. XPS spectra for hydrogel groups.

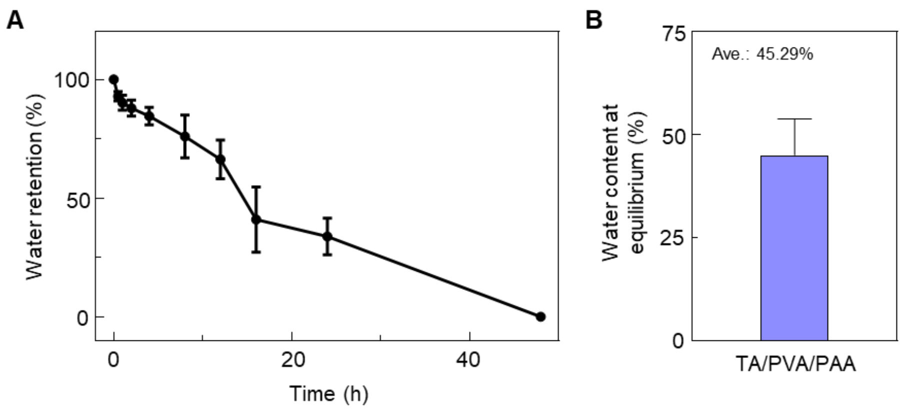

Figure S2. (A) Water retention-time profile and (B) water retention at equilibrium of TA/PVA/PAA hydrogel (n = 9, for each group).

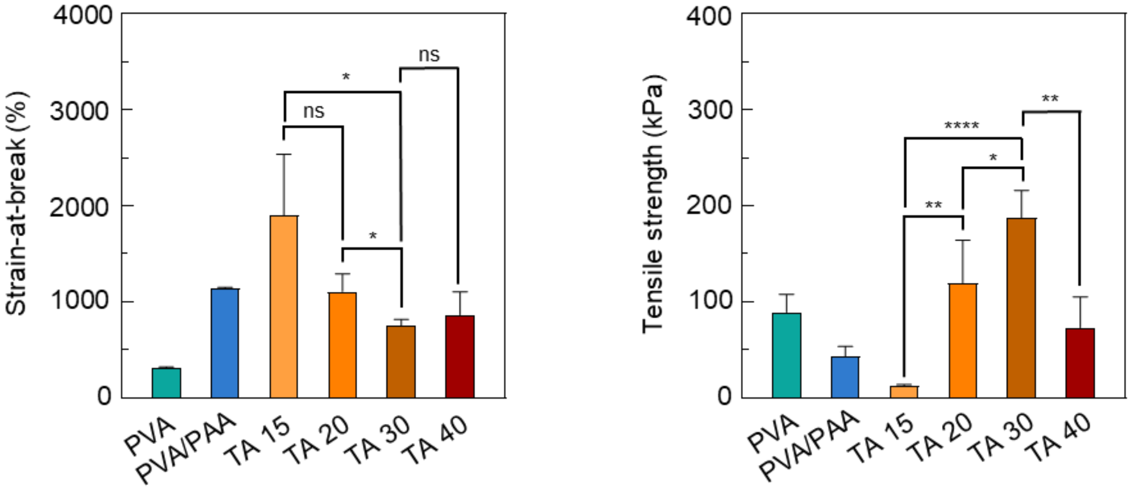

Figure S3. Strain-at-break and maximum tensile strength of PVA, PVA/PAA, and TA/PVA/PAA hydrogels. TA 20 denotes the TA/PVA/PAA hydrogel with a TA concentration of 20% w/v (n = 3, for each group).

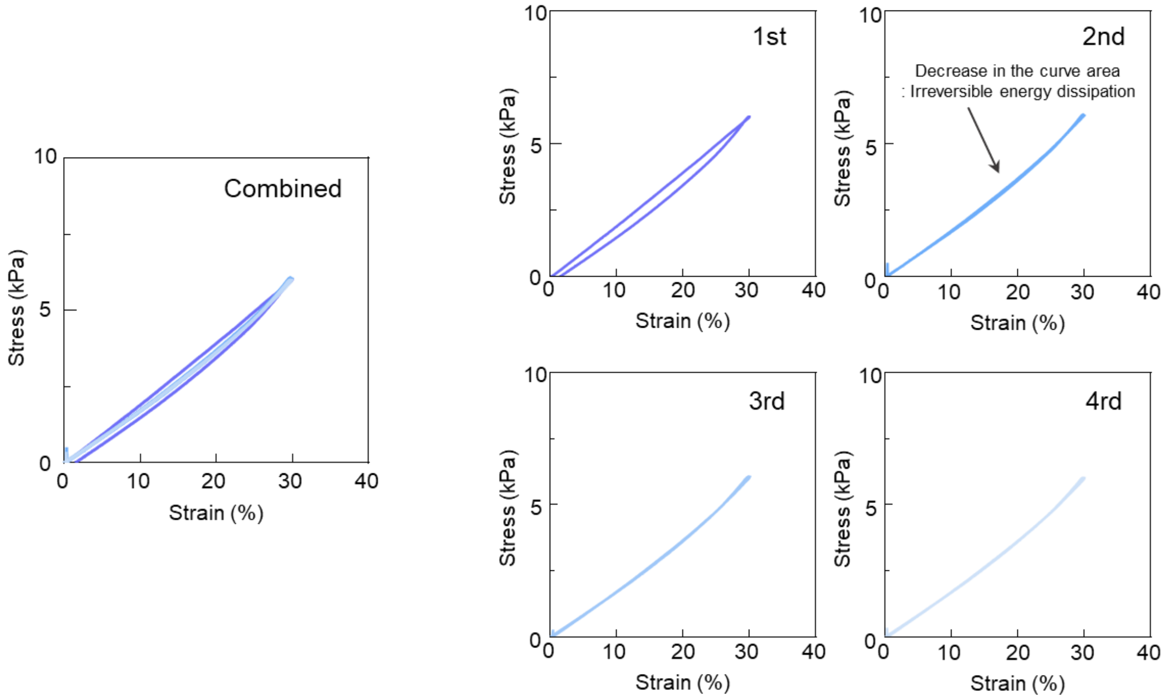

Figure S4. Cyclic loading-unloading test for the PVA/PAA hydrogel.

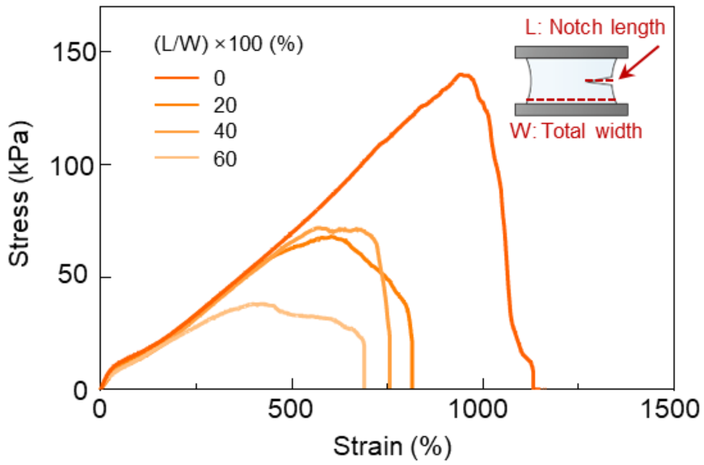

Figure S5. Tensile test curves of the hydrogels with varying notch lengths.

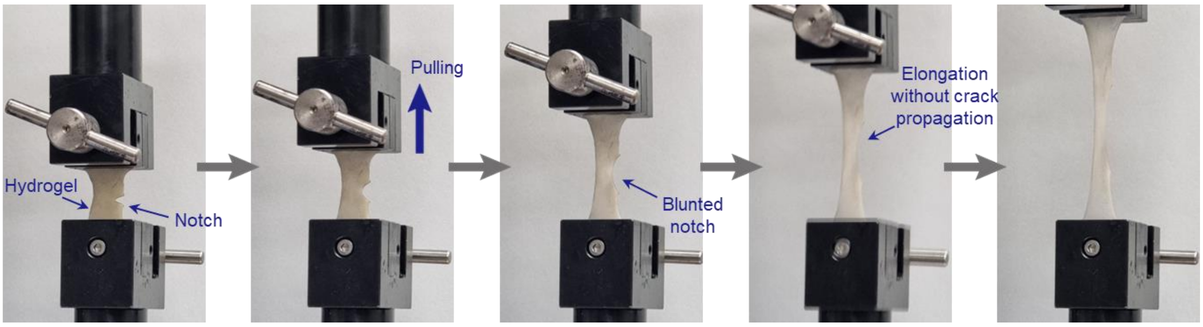

Figure S6. Sequential image showing elongation of the notch-introduced hydrogel.

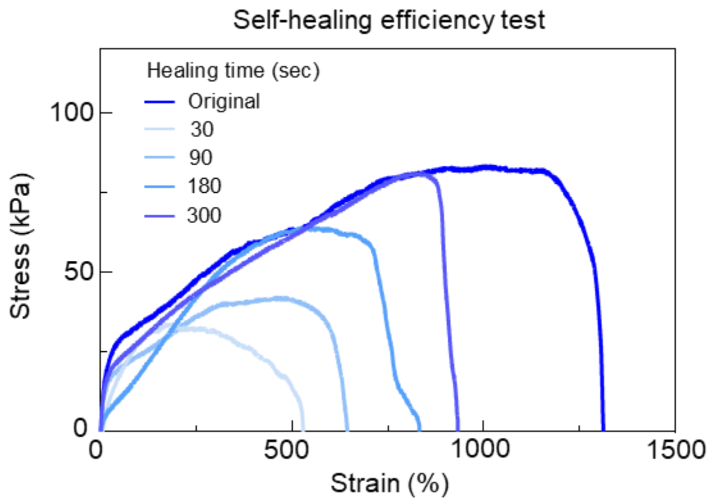

Figure S7. Tensile test curves for the self-healed hydrogels with varying healing times.

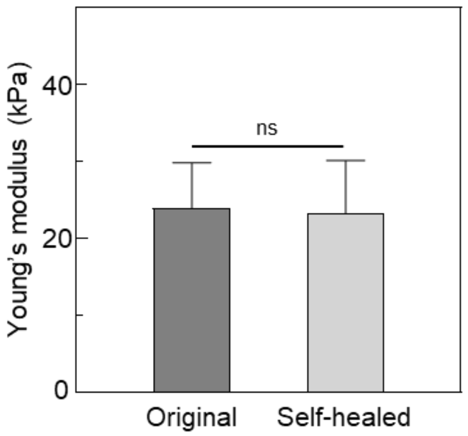

Figure S8. Young's moduli of TA/PVA/PAA hydrogel before and after the self-healing. (n = 3, for each group)

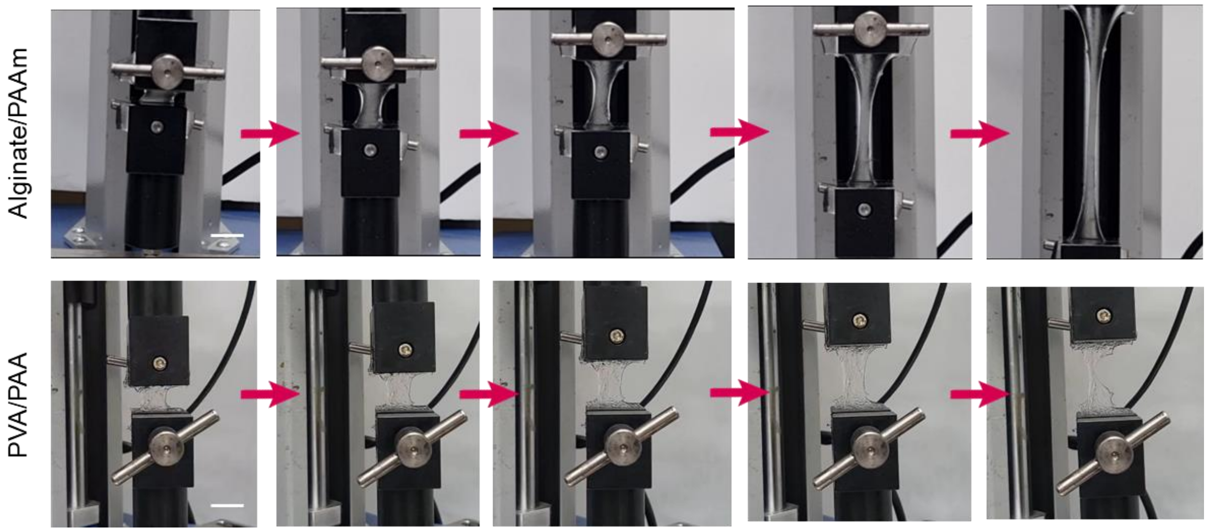

Figure S9. Sequential photographs showing stretching of Alginate/PAAm and PVA/PAA hydrogels (scale bars, 20 mm).

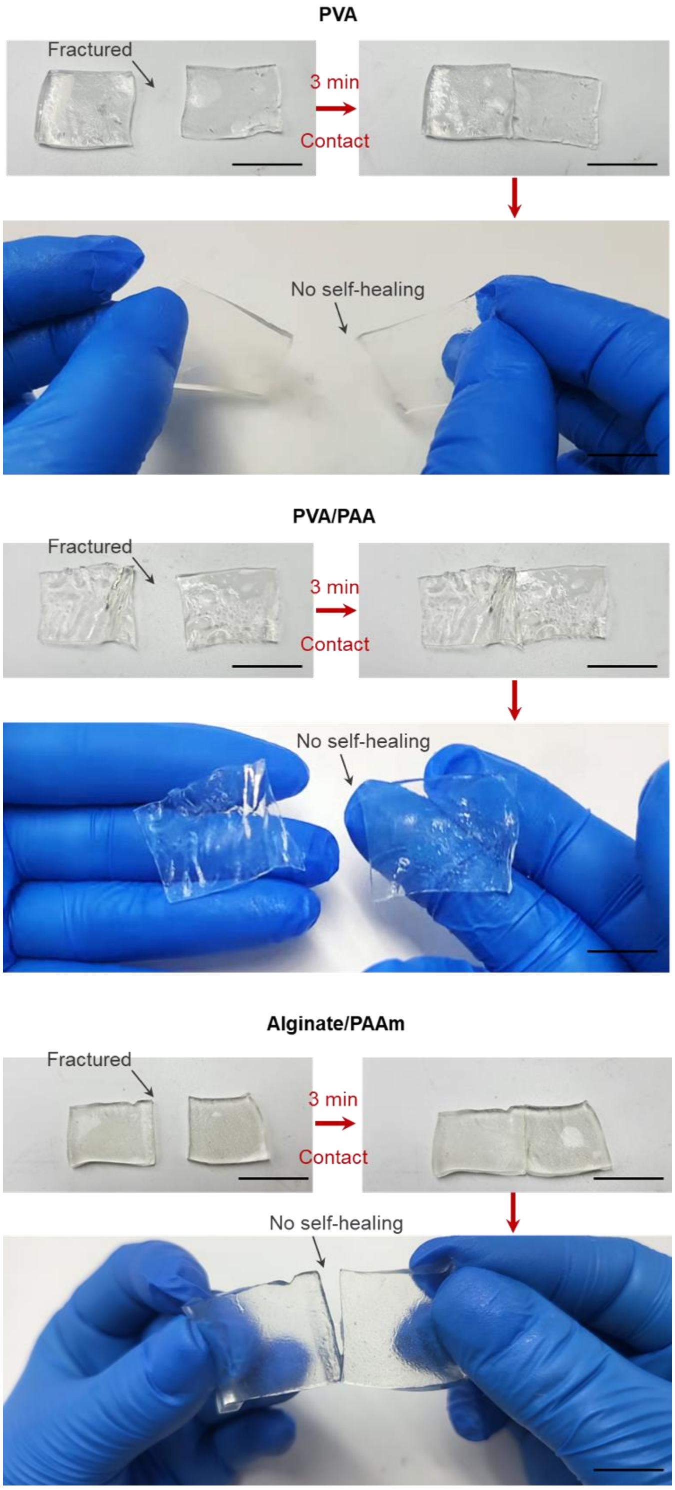

Figure S10. Photographs of PVA, PVA/PAA, and alginate/PAAm hydrogels showing their lack of self-heal ability.

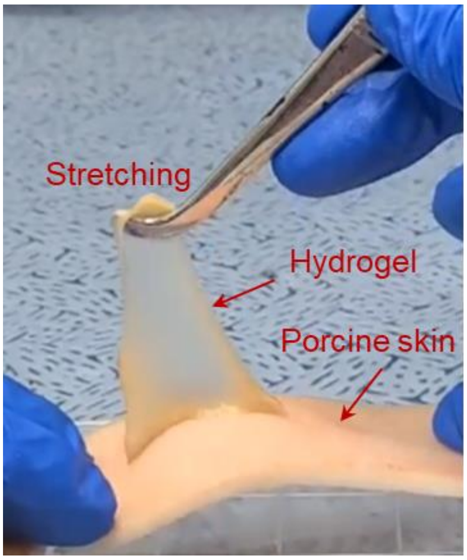

Figure S11. Optical image of the hydrogel tough-bonded to a porcine skin surface.

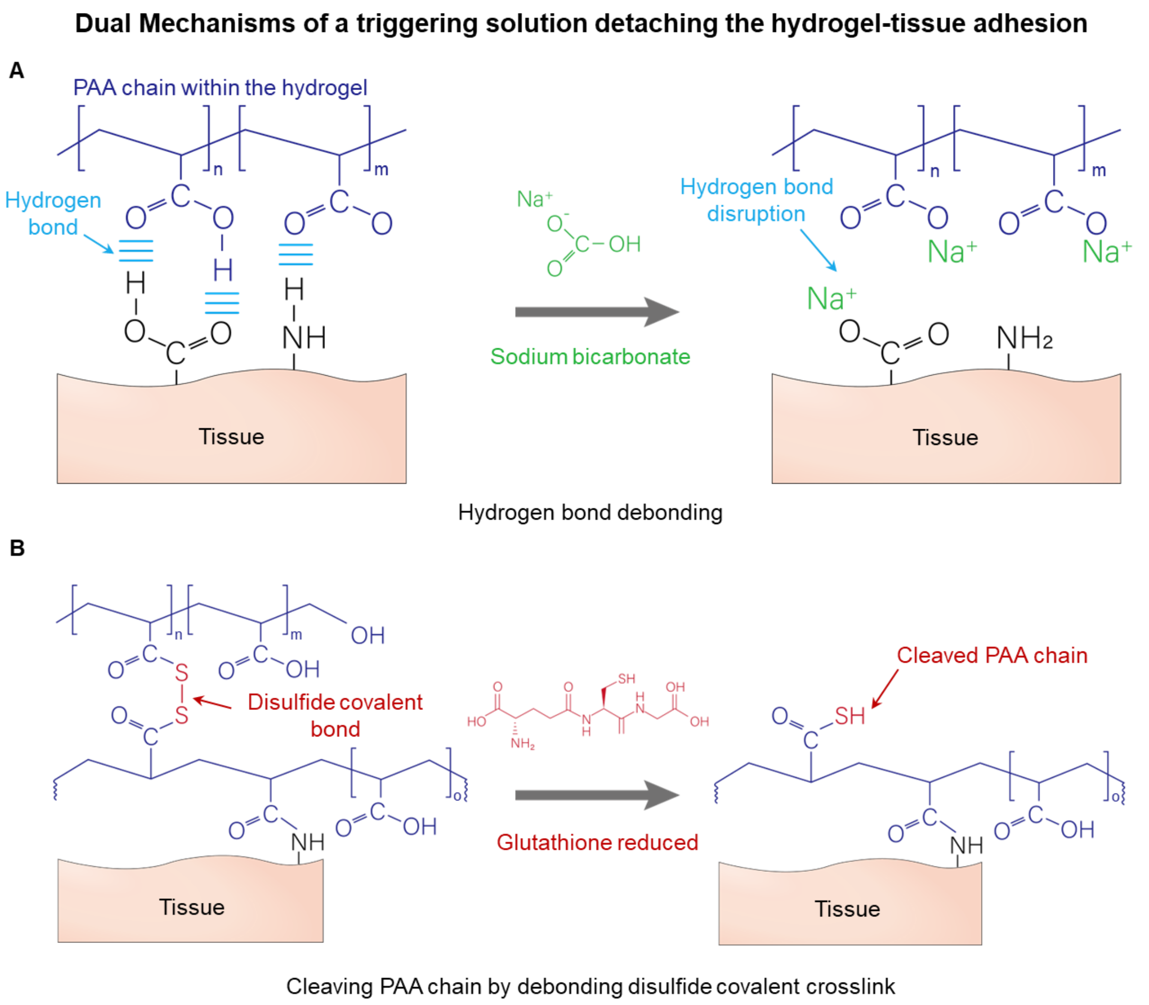

Figure S12. Schematic illustration of the triggering solution detaching the hydrogel from tissue surfaces.

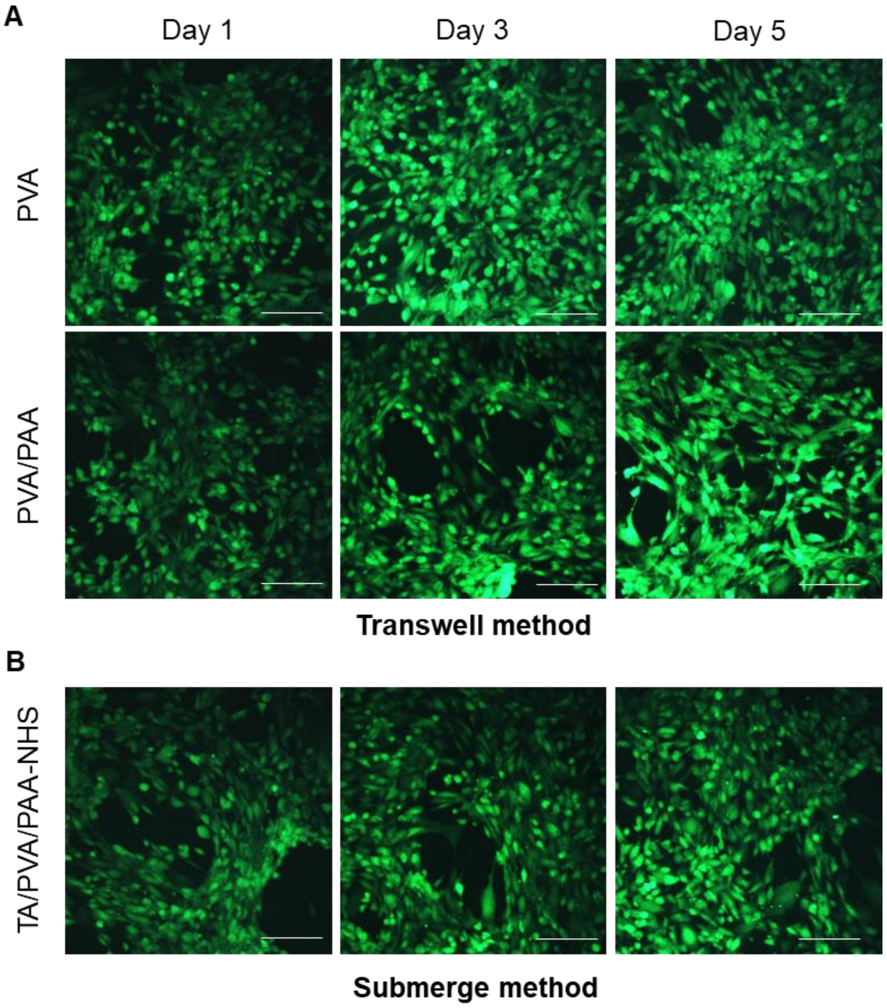

Figure S13. *In vitro* biocompatibility tests on hydrogels. (A) Fluorescence microscopy images of live/dead stainings (green: live, red: dead) on NIH 3T3 cells cultured with PVA and PVA/PAA hydrogels through a transwell system (scale bars, 100 μm). (B) Fluorescence images of live/dead stainings on NIH 3T3 cells directly cultured with submerged TA/PVA/PAA-NHS hydrogel (scale bars, 100 μm).

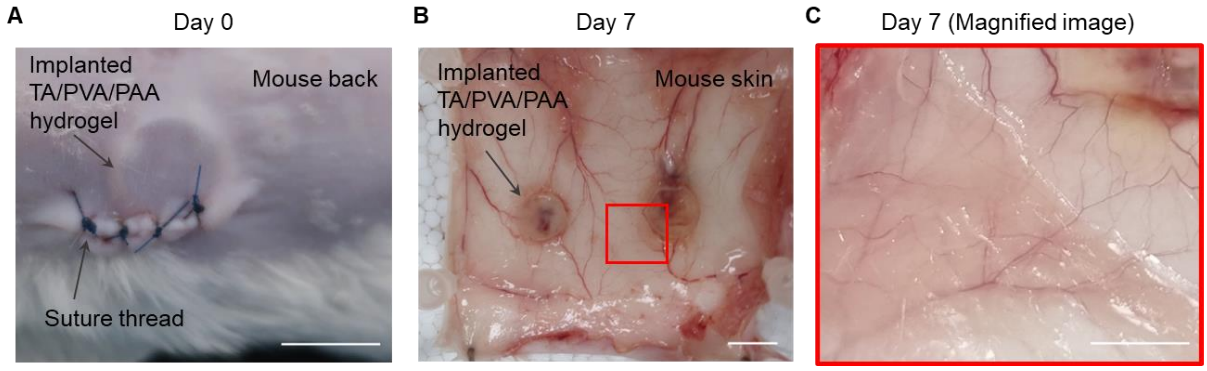

Figure S14. Optical photographs of the hydrogel implanted into a mouse subcutaneous skin. (A) The subcutaneously implanted TA/PVA/PAA hydrogel right after the implantation (scale bar, 0.5 cm). (B) The subcutaneous skin tissue near implanted TA/PVA/PAA hydrogel after seven days of the implantation (scale bar, 5 mm). (C) Magnified photograph image of the subcutaneous skin tissue adjacent to the implanted hydrogel (scale bar: 5 mm)

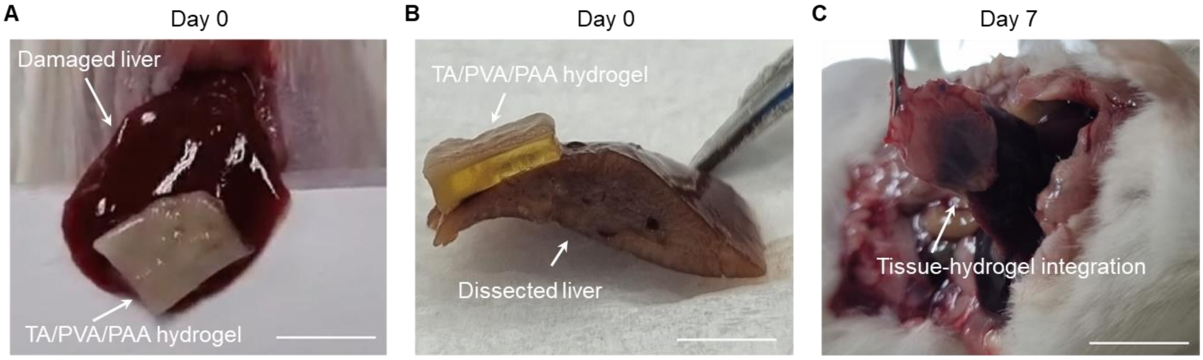

Figure S15. Photographs of the TA/PVA/PAA hydrogel adhered to liver tissue. (A) The TA/PVA/PAA hydrogel covering the bleeding site of the liver (scale bar, 5 mm). (B) The dissected hydrogel-attached liver (scale bar, 5 mm). (C) The hydrogel integrated with the liver tissue after seven days of the application (scale bar, 5 mm).

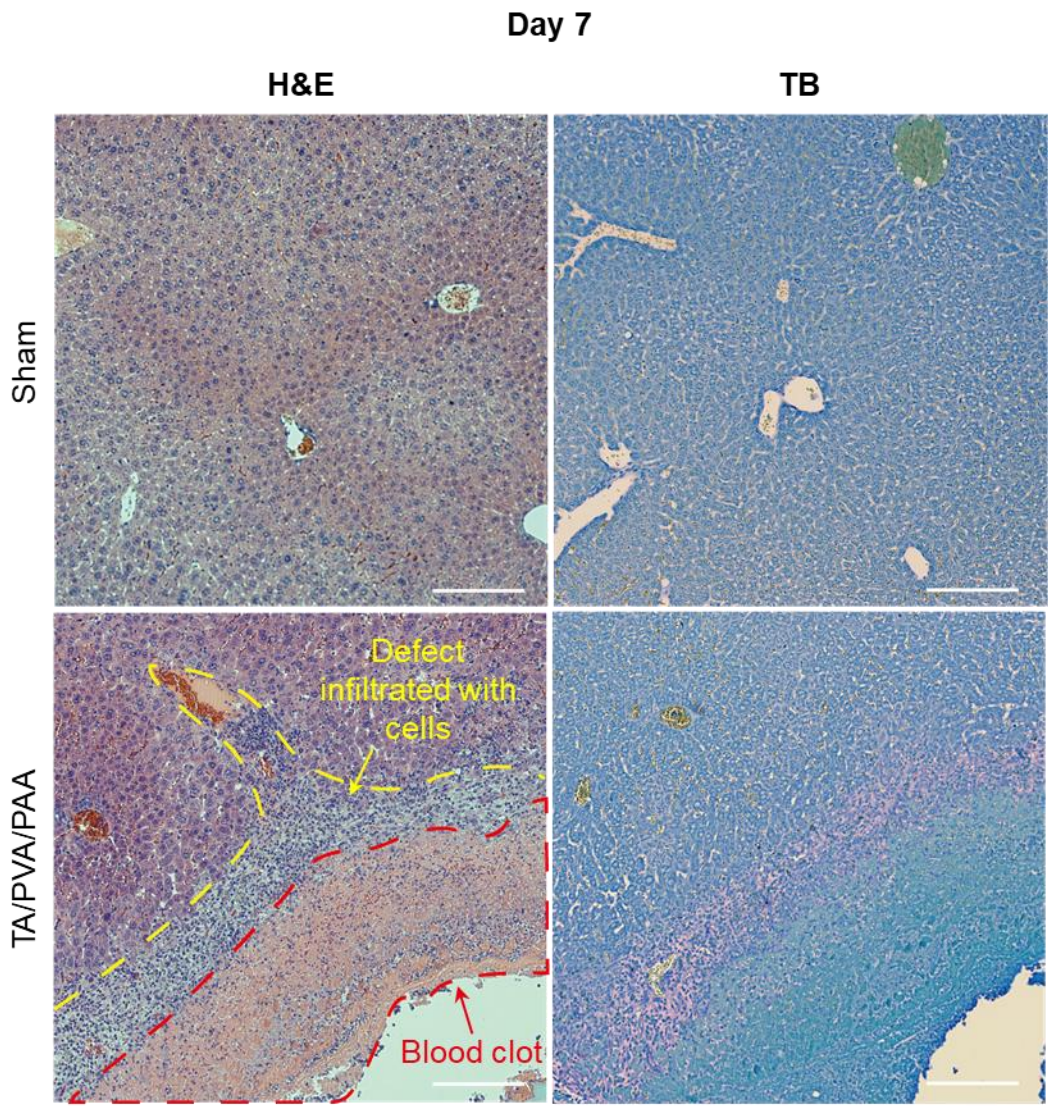

Figure S16. Histological images of the liver tissue of the sham group and where the TA/PVA/PAA group (scale bar: 200 µm). For the TA/PVA/PAA group, the tissue site where hemostasis was induced was stained. A yellow dashed line indicates a defect region that was formed by a needle puncture. A red dashed line signifies a blood clot structure.
